# Meta-Analysis of the Relationship between Multiple Sclerosis and Migraine

**DOI:** 10.1371/journal.pone.0045295

**Published:** 2012-09-14

**Authors:** Julia Pakpoor, Adam E. Handel, Gavin Giovannoni, Ruth Dobson, Sreeram V. Ramagopalan

**Affiliations:** 1 Wellcome Trust Centre for Human Genetics, University of Oxford, Roosevelt Drive, Headington, Oxford, United Kingdom; 2 Nuffield Department of Clinical Neurosciences (Clinical Neurology), University of Oxford, The West Wing, John Radcliffe Hospital, Oxford, United Kingdom; 3 Blizard Institute, Queen Mary University of London, Barts and The London School of Medicine and Dentistry, London, United Kingdom; 4 London School of Hygiene and Tropical Medicine, London, United Kingdom; Innsbruck Medical University, Austria

## Abstract

**Background:**

Studies investigating a proposed association between multiple sclerosis (MS) and migraine have produced conflicting results and a great range in the prevalence rate of migraine in MS patients. By meta-analysing all available data we aimed to establish an overall estimate of any association in order to more accurately inform clinicians and care-givers about a potential association between MS and migraine.

**Methods:**

Pubmed and EMBASE were searched to identify suitable studies. Studies were included if they were a case-control study or cohort study in which controls were not reported to have another neurological condition, were available in English, and specified migraine as a headache sub-type. The odds ratio (OR) of migraine in MS patients vs. controls was calculated using the inverse variance with random effects model in Review Manager 5.1.

**Results:**

Eight studies were selected for inclusion, yielding a total of 1864 MS patients and 261563 control subjects. We found a significant association between migraine and MS (OR = 2.60, 95% CI 1.12–6.04), although there was significant heterogeneity. Sensitivity analysis showed that migraine without aura was associated with MS OR = 2.29 (95% CI 1.14–4.58), with no significant heterogeneity.

**Conclusions:**

MS patients are more than twice as likely to report migraine as controls. Care providers should be alerted to ask MS patients about migraine in order to treat it and potentially improve quality of life. Future work should further investigate the temporal relationship of this association and relationship to the clinical characteristics of MS.

## Introduction

Multiple sclerosis (MS) is an inflammatory disease of the central nervous system (CNS) characterized by myelin loss, varying degrees of axonal pathology, and progressive neurological dysfunction. The clinical features of MS encompass an extremely wide range of neurological symptoms but migraine is not typically included [1]. Migraine is a common chronic debilitating condition, with an estimated 1-year period prevalence of 11.7%, which reduces the quality of life in many sufferers [2], [3].

Within Europe, migraine has a high economic impact, with headaches (including migraine) estimated to cost €43.5 billion per capita in 2010 [4]. MS and migraine have a number of demographic similarities including a female preponderance and relatively young age at onset [5], [6]. Epidemiological similarities also exist, as both conditions have a higher prevalence in Caucasian as compared to African or Asian populations [7], [8]. In 1952 Compston and McAlpine found that 2% of MS sufferers experienced migraine within 3 months of MS onset [9]. This finding stimulated further studies but limitations, including small sample sizes, has resulted in conflicting results and a great range in the prevalence rate of migraine in MS patients [10]–[18]. In this meta-analysis we sought to provide an overall estimate of the relationship between MS and migraine by comparing the occurrence of migraine in MS patients vs. controls in order to accurately inform clinicians.

**Table 1:** Information about the included studies.

First author & year of publication	Type ofstudy	MS diagnostic criteria	Migraine diagnostic criteria	Cases	Controls	OR (95% CI)	F/M ratio	Case source	Control source	Age range and mean age of MS patients (years)	MSsubtype	Mean MS duration (years)	Median EDSS score	Migraine ascertainment
Watkins 1969	Case control	Definite/probable MSMcAlpine criteria	Critchley 1967	100	100	2.71 (21.28–5.73)	1.78	Hospital (prevalent)	Hospital	Range 15–50	N/a	N/a	N/a	Interview
Zorzon 2003	Case-control	Definite MS McDonald criteria	N/a	140	131	20.41 (2.69–154.82)	1.8	Multiple Sclerosis Center (prevalent)	Blood Transfusion Center	Range 17–65, mean 42.1	71.4% RRMS,19.3% SPMS 9.3% PPMS	10.9	2	Questionnaire in interview
Vacca 2007	Case-control	Definite MS McDonald criteria	InternationalClassification of Headache Disorders 2004 (2nd edition)	238	238	3.19 (2.11–4.85)	1.9	Hospital (prevalent)	Friends	Range 24–61	75.6% RRMS 24.4% SPMS	n/a	2.5	Interview
Nicoletti 2008	Case-control	Definite/probable MS, Poser criteria	International Headache Society criteria, 1988	101	101	1.31 (0.64–2.71)	1.81	Cohort (incidence and prevalence)	Cohort	Mean 43.6	75.2 RRMS19.0 SPMS2.0 PPMS	9.9	N/a	Standardised questionnaire in interview. If doubtful diagnosis, underwent neurological examination
Putzki 2009	Case-control	Definite MS Poser or McDonald criteria	International Headache Society criteria	491	447	0.49 (0.37–0.65)	2.13	Hospital (prevalent)	Cohort^a^	Mean 45.3	63.7 RRMS20.2 SPMS9.2 PPMS5.1% uncertain	11.5	Median n/a, EDSS mean 3.17	Validated questionnaire
Kister 2010	Case-control	Definite MS McDonald criteria	International Classification of HeadacheDisorders, 2^nd^ edition	204	162576	6.47 (4.91–8.52)	N/a	MS Care Center (prevalent)	Cohort^a^	Mean 45	N/a	12.5	N/a	Validated questionnaire
Katsiari 2011	Case-control	Definite MSMcDonald criteria	ICHD-II, 2004	48	72	1.04 (0.43–2.49)	25.32	Hospital (prevalent)	Hospital	Mean 37.8	N/a	7.5	|Median n/a, EDSS mean 3.0 at the 1 year follow up	Standardised questionnaire in interview. If positive response, underwent neurologic examination
Kister 2012	Cohort	Definite/probable MS Poser or McDonald criteria	Physician-diagnosed migraine	542	97898	5.65 (4.68–6.82)	All female	Cohort (incident & prevalent)	Cohort	Range 25–42^b^	N/a	N/a	N/a	Questionnaire/medical record

OR (95%CI) = odds ratio with 95% confidence intervals, F = female, M = male, N/a = not available, RRMS = relapsing-remitting MS, SPMS = secondary-progressive MS, PPMS = primary progressive MS.

a Historical controls used.

b Age range of all individuals in the Nurses’ Health Study II.

## Methods

### Article Search

Pubmed was searched by JP and AEH for abstracts using the terms (“multiple sclerosis”[MeSH Terms] OR (“multiple”[All Fields] AND “sclerosis”[All Fields]) OR “multiple sclerosis”[All Fields]) AND (“migraine disorders”[MeSH Terms] OR (“migraine”[All Fields] AND “disorders”[All Fields]) OR “migraine disorders”[All Fields] OR “migraine”[All Fields]) and (“multiple sclerosis”[MeSH Terms] OR (“multiple”[All Fields] AND “sclerosis”[All Fields]) OR “multiple sclerosis”[All Fields]) AND (“headache”[MeSH Terms] OR “headache”[All Fields]). EMBASE was searched for abstracts using the terms “multiple sclerosis [All fields] AND migraine [All fields]. No limitations or time period restrictions were applied; the latest search was undertaken on the 17^th^ December 2011. We were not familiar with any study currently in progress to be considered for inclusion. Published conference abstracts were eligible for inclusion and so were both prospective and retrospective studies. Studies were subsequently excluded if they were not a case-control study or cohort study, if the article was not available in English, or if migraine was not specified as a headache sub-type. Studies where controls had other neurological conditions were also excluded. The abstracts of the resulting articles were hand-searched in order to select studies. Attempts to identify further articles were done by searching the references of the studies. Data on study type, raw numbers of MS patients and controls who had and had not experienced migraine and their sex; diagnostic criteria of MS and migraine used; method of migraine ascertainment; source of cases and controls; age range and mean age of patients and controls; MS patient subtype, mean diseases duration and median EDSS score was extracted independently from included articles. Any discrepancy on the suitability for inclusion of a study between the authors was resolved by consulting a third author (SVR).

**Figure 1:**
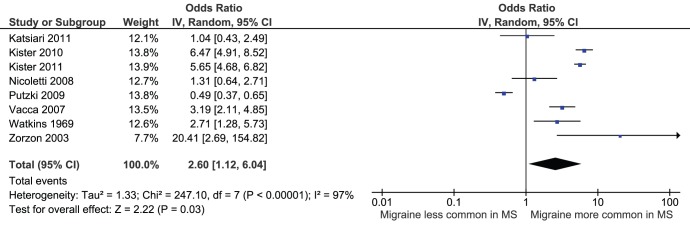
Forest plot of comparison: MS patients vs. control, outcome: migraine.

### Statistical Analysis

**Figure 2:**
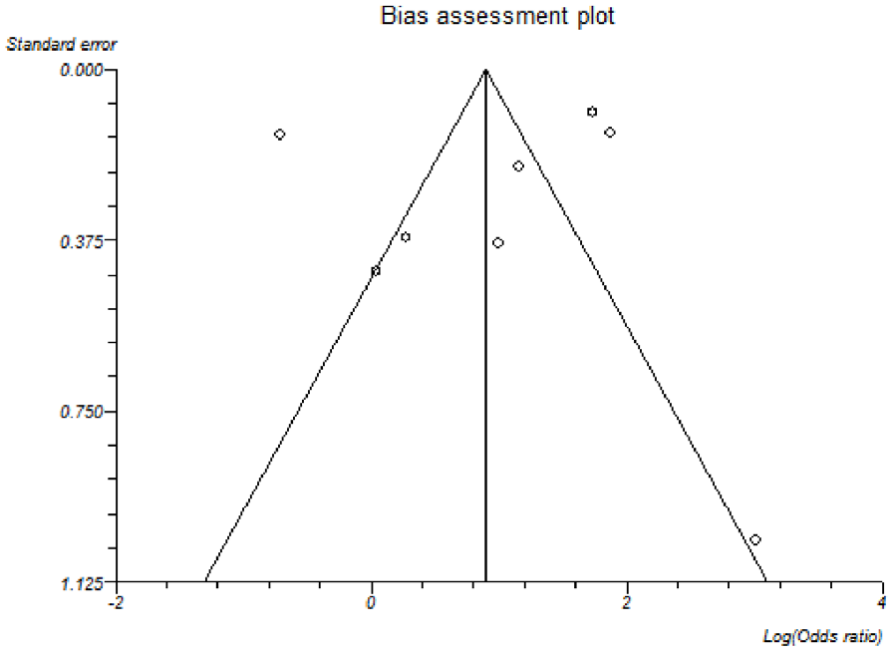
Funnel plot of included studies.

The inverse variance model in Review Manager 5.1 was used to calculate the overall odds ratio (OR), 95% confidence interval (95% CI) and test statistic for the relationship. Statistical significance was set at p<0.05. Statistical heterogeneity of studies was assessed through the calculation of Tau^2^ and I^2^. A random effects model was applied unless the I^2^ was ≤25% in which case a fixed effects model would be used [19]. In attempting to dissipate any heterogeneity, sensitivity analysis was done by excluding individual studies to see if there was a notable reduction in heterogeneity and further, subgroup analysis was performed on studies which differentiated between migraine with and without aura. Generation of a funnel plot and the Egger p-value allowed determination of the potential publication bias of included studies. Further, the quality of the studies was assessed using the Newcastle-Ottawa Scale (NOS).

## Results

### Included Studies

Our Pubmed search initially yielded 654 studies (of which some studies appeared under more than one search term) and the EMBASE search 705 studies. In total, 8 studies were selected for inclusion according to the described inclusion criteria [11]–[18], yielding a total of 1864 MS patients and 261563 control subjects. However, it should be noted that Kister et al 2010 and Nicoletti et al 2008 used historical controls [15], [18]. Information about the included studies can be found in **table 1** and the assessed quality of each study using NOS is indicated in **table 2**.

**Table 2:** Newcastle-Ottawa Scale (NOS) assessment of the quality of the studies.

Study	Selection (max 4 ✓)	Comparability (max 2 ✓)	Exposure (max 3 ✓)
Katsiari 2011	✓✓	✓✓	✓
Kister 2010	✓✓	✓✓	✓
Kister 2012	✓✓✓✓	✓✓	✓✓
Nicoletti 2008	✓✓✓	✓✓	✓
Putzki 2009	✓✓✓	✓✓	✓
Vacca 2007	✓✓✓✓	✓✓	✓
Watkins 1969	✓✓✓	✓✓	✓✓
Zorzon 2003	✓✓✓✓	✓✓	✓✓

**Figure 3:**
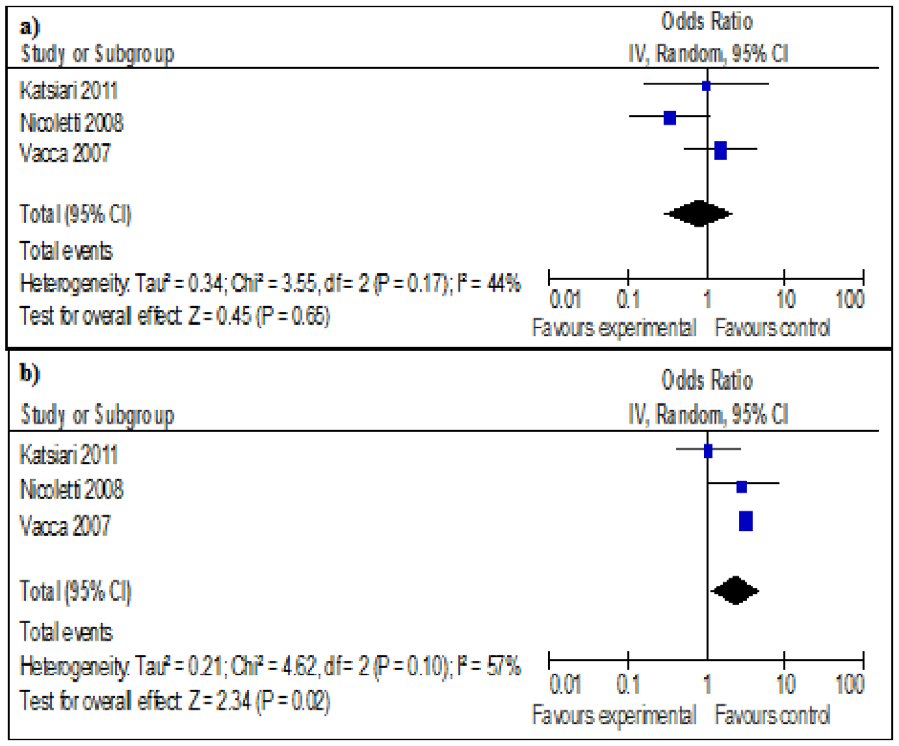
Forest plot of comparison: MS patients vs. control.

### Migraine in MS Patients vs. Control

Migraine was more common in MS patients than controls. The overall OR upon inclusion of all 8 studies was 2.60 (95% CI 1.12–6.04), **fig 1**, however, significant statistical heterogeneity was identified (I^2^ = 97% Chi^2^ = 247.10, P<0.00001). Publication bias assessment is shown using a funnel plot, **fig 2** **.** No selective reporting of outcomes was found for any study when comparing methods of studies to results. There was no significant indication of publication bias, Egger p-value = 0.64. The results of subgroup analyses of studies investigating migraine with and without aura showed a significant co-morbid association between MS and migraine without aura (OR = 2.29 (CI = 1.14–4.58)) without any significant heterogeneity (**Table 3** **and** **Figure 3**).

**Table 3:** Odds ratio and 95% confidence intervals for subgroup studies.

Subgroup of studies	Odds ratio with 95% CI	P-value	Number of studies	Heterogeneity
Migraine with aura	0.8 (0.29–2.16)	P = 0.65	3[16]–[18]	Tau^2^ = 0.34; Chi^2^ = 3.55, df = 2 (P = 0.17); I^2^ = 44%
Migraine without aura	2.29 (1.14–4.58)	P = 0.02	3[16]–[18]	Tau^2^ = 0.21; Chi^2^ = 4.62, df = 2 (P = 0.10); I^2^ = 57%

Abbreviations: CI = confidence interval, df = degree of freedom. Tau^2^ estimates the between-study variance and I^2^ describes the proportion of variation estimated to be due to heterogeneity.

## Discussion

This meta-analysis has shown that there is a significant association between migraine and MS, with MS patients being more than twice as likely to report migraine as controls. Limitations of this study include the nature of the studies included and study heterogeneity [20]. Of note, in subgroup analyses, migraine without aura was significantly increased in patients with MS (OR = 2.29 (CI = 1.14–4.58)) without any significant heterogeneity. The heterogeneity may be in part explained by variation in the demographic characteristics (as shown in **table 1** **),** recall/reporting/interviewer bias of migraine in some studies as this data was largely collected by questionnaire/interview and ascertainment/selection bias of MS patients and controls. As indicated in **Table 2** **,** we found some discrepancy in the quality of the studies and the potential for bias. Bias may result in an over or under-estimation of ORs depending on whether the bias is differential or non-differential with respect to MS patients and controls. It can be speculated that MS patients are more likely to be in contact with a neurologist and may thus be more likely to report migraine and/or have migraine detected, resulting in an overestimated OR. Similarly, an MS patient may be more attentive to the frequency and severity of migraine.

Whereas the occurrence of migraine without aura but not migraine with aura was found to be significantly more frequent in MS patients, it is plausible that small sample sizes in studies investigating migraine with aura meant there was insufficient power to detect significance.

Further, the study by Rolak and Brown, which was not included in this investigation as the controls had neurological disorders, found migraine reported in 22/104 MS patients vs. in 10/100 control neurological patients, OR 2.41 (CI 1.08- 5.40) [10].

The mechanism behind any association between MS and migraine remains to be determined but a number of hypotheses exist. Nicoletti et al compared the age of onset of both conditions which was 33.6 -+10.8 years for MS and 19.5+- 7.4 years for migraine [18]. Since migraine often precedes MS by numerous years, as also shown by Kister et al, another potential explanation is that migraine could be a potential risk factor for MS [11]. However, cortical demyelination has been shown to accelerate cortical spreading depression in rodent models of autoimmune induced cortical demyelination, a key aspect in migraine pathophysiology [21]. Migraine may therefore result from early MS lesions. Importance of lesion location in MS patients has also been suggested following observations that lesions within the midbrain are more commonly associated with co-morbid migraine in MS patients. This may be explained by the presence of the periaqueductal gray matter (PAG) within the midbrain which is suspected to have a role in migraine aetiology [22], [23].

Immune therapies used to treat MS may also a have a role as evidence for a migraine-inducing role of interferon beta exists [24], however, immune therapies could only ever provide a small part of the complete explanation due to the fact that migraine often precedes MS. Interferon beta treatment is also thought to worsen pre-existing migraine, suggesting it may not be implicated in the aetiology of the migraine itself [25]. Notably, evidence suggests that the other disease modifying therapies glatiramer acetate and natalizumab do not appear to worsen migraine, and indeed some patients may want to take this into consideration as switching from interferon beta may help reduce both frequency and severity of migraine [26]–[27]. Furthermore, stress and anxiety, which often accompany MS, are thought to be very likely causes of increased migraine frequency and chronification [28]. One further suggestion is that instead of one condition causing the other, both conditions have a common pathway in their underlying cause.

Before any of the above hypotheses can be confirmed, more studies specifically investigating whether MS precedes migraine or vice versa would be necessary to determine the temporal relationship of the demonstrated association and clarify whether migraine is as a risk-factor, co-morbidity or symptom of MS. Regardless, the key aspect of this study is the potentially underappreciated finding of migraine being more common in MS patients and as a major potential cause of poorer quality of life, it should be actively looked for and treated.
