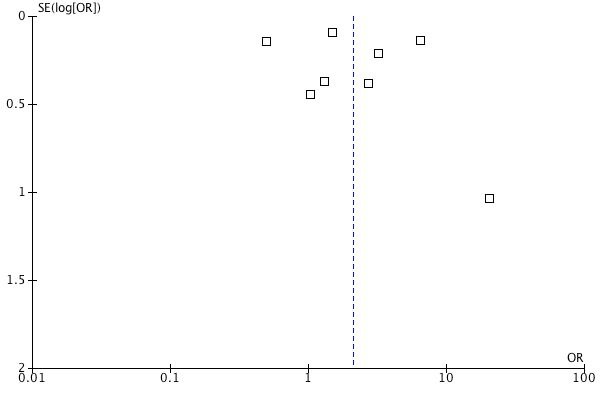# Correction: Meta-Analysis of the Relationship between Multiple Sclerosis and Migraine

**DOI:** 10.1371/annotation/274e5ef1-79aa-48f7-af62-0594d5c1354e

**Published:** 2013-04-01

**Authors:** Julia Pakpoor, Adam E. Handel, Gavin Giovannoni, Ruth Dobson, Sreeram V. Ramagopalan

It has come to the attention of the authors that the value we included for the number of controls participating in the paper by Kister et al 2012 (2011 as epub) in our study “Meta-analysis of the Relationship between Multple Sclerosis and Migraine” is inaccurate.

Corrected values for Kister 2012 for Table 1:

Total controls: 115888

Controls with migraine: 24337

Controls without migraine: 91551

Fig. 1: 

**Figure:**
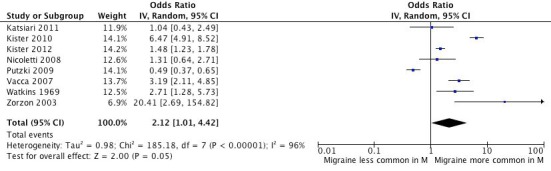


Fig. 2: 

**Figure:**